# The effect of social geographic factors on the untreated tooth decay among head start children

**DOI:** 10.4317/jced.54228

**Published:** 2017-10-01

**Authors:** Masahiro Heima, Margaret Ferretti, Mehveen Qureshi, Gerald Ferretti

**Affiliations:** 1 Assistant Professor, Department of Pediatric Dentistry, School of Dental Medicine, Case Western Reserve University; 2 Assistant Professor, Department of Pediatric Dentistry, School of Dental Medicine, Case Western Reserve University; 3 School of Dental Medicine, Case Western Reserve University; 4 Professor, Department of Pediatric Dentistry, School of Dental Medicine, Case Western Reserve University

## Abstract

**Background:**

Disparities among untreated dental caries exist for children from low-income families in the United States. Understanding of the mechanism of the disparities is required to reduce it and social geographic factors are one of the important influences. Although the effect of fluoridated water has been well reported, studies of other sociogeograpic factors, such as the density of available dentists, are still very limited. The objective of this study is to explore the effect of sociogeographic factors on the number of primary teeth with untreated dental caries among children from low-income families who are enrolled in Head Start programs throughout Northeast Ohio of the United States.

**Material and Methods:**

This was a cross-sectional chart review study. Three hundred-eighty-eight charts were reviewed, and the number of primary teeth with untreated dental caries (dt) and the children’s addresses were retrieved. The sociogeographic variables, including fluoridated water availability and the density of available dentists who accept a government-supported insurance (Medicaid dentists), were collected.

**Results:**

The mean (standard deviation) of children’s age was 3.51 (1.14) years with a range of 7 months to 5 years. A negative binomial regression model analysis, which used dt as a dependent variable and children’s characteristic factors (i.e. age, gender, insurance type, and total number of primary teeth) and sociogeographic factors (i.e. Population, total number of Medicaid dentists, density of Medicaid dentist, and Fluoride water availability) of cities, as independent variables, demonstrated that only the density of Medicaid dentist in the sociogeographic factors indicated a significant effect (Estimated ß-Coefficients (Standard Errors)=-0.003 (0.002), *p*=0.030).

**Conclusions:**

This study demonstrated a significant negative association between the density of available dentists and untreated dental caries among children from low-income families in Head Start programs in Northeast Ohio. Increasing available dentists may be a strategy to reduce the number of early childhood caries.

** Key words:**Child, poverty, dental caries, Health Services Accessibility.

## Introduction

Dental caries is the most common chronic childhood disease in the United States of America (i.e. U.S.) (1). The prevalence of dental caries among 2-5 year old U.S. children was 24% in 1988-1994 (2). A recent estimate still indicated 23% in 2011-2012 (3). An especially higher prevalence of decay has been observed among children from low-income and or minority families (2,3). About 42% of children (2-5 years) from low-income families compared to 18% of children from non-low-income families had dental caries in their primary teeth (2). Twice as many non-Hispanic black children had untreated dental caries (21%) when compared to non-Hispanic white children (10%) (3).

Children’s demographic characteristics, such as low-income, minority race, or being children of low educational parents, impact the number of children’s dental caries mediated by dental utilization (4,5); if the children have dental access even with high caries risk due to their demographic characteristics, they have fewer dental caries. In the U.S., a governmental supported health insurance (Medicaid) is available for children from low-income families. Although all treatment cost for dental care is covered through Medicaid, dental utilization by the children is not adequate. The median dental utilization rates are only 33-37% across the U.S. (6,7). This inadequate dental utilization is caused by complex problems, including difficulties with locating a dentist who accepts these children, lack of transportation, caregiver’s oral health belief and dental experiences, and the dentist’s perception of children with Medicaid (7-11).

A framework by Fisher-Owens (12) describes multiple stages of influence on children’s dental caries that involves a “child’s level” to a “community level”. A comparatively fewer articles about community-level influence, which can be a community level of barriers, have been published than family and child levels. Understanding the community-level influence on children’s dental caries may help reduce the oral health disparity (2).

One of the major barriers is finding a dentist who accepts children with Medicaid (9-11). Many children enrolled in Medicaid fail to receive needed dental care because a dental provider refuses to accept Medicaid based on low reimbursement rates and lower patients’ compliance (7,13). For example, only 7% of general dentists and 29% of pediatric dentists around this research area, the state of Ohio, accept children who are Medicaid recipients (14). Even in instances where a dental clinic is next door, there is no guarantee that a child from a low-income family will have access.

Research regarding community-level influence, including sociogeographic factors, (8,9,11,15) (i.e the density of dentists) has indicated that there is a significant positive relationship between the density of dentists who accept Medicaid children (the number of pediatric dentists per 10,000 Medicaid-enrolled children) and the number of Medicaid-enrolled children who utilize dental care. However, research regarding community-level influence on children’s oral health status, such as the number of untreated dental caries, is very limited.

Another important sociogeographic factor is fluoridated tap water availability. Fluoridated water is one of the most well-known effective ways to prevent children’s dental caries. Previous study among children from low-income families indicated that children who had access to fluoridated water showed less dental caries than in children who did not have access to fluoridated water (16-18).

The Head Start/Early Head Start Programs (HS program) are federally funded from the United States Department of Health and Human Services. HS programs support the comprehensive development of children from birth to age 5 and include early learning, as well as health and family well-being (19). The Department of Pediatric Dentistry at Case Western Reserve University provides dental services to children from HS programs in the Northeast Ohio state area through dental examination, prophylaxis, and 5% fluoride varnish application. According to multiple studies regarding oral health and dental accessibility among children who were enrolled in Head Start program, it was reported that these children face high risk of dental caries (10). However, the solutions for solve the high untreated dental caries rate among Head Start children are not yet clear.

The objective of this study is to explore the effect of sociogeographic factors among children from low-income families enrolled in the HS program throughout Northeast Ohio. We tested our hypothesis that the dentist density and fluoride water availableness negatively associated with children’s untreated dental caries among low-income families in Northeast Ohio.

## Material and Methods

-Method

This is a cross-sectional chart review study. Charts generated from January 2011 to December 2014 were reviewed. Head Start sites were located throughout Northeast Ohio, which the Department of Pediatric Dentistry at Case Western Reserve University had been serving since 2007. The sample size has been calculated based on the total number of charts (5696 charts), 5% confidence interval and 95% confidence level in order to generate a representative sample of Northeast Ohio. We aimed for 360 charts, and this included 10% of charts that would be excluded. 400 charts were randomly selected for the review. All research information was extracted from the first dental examination at the Head Start site. Standardized dental caries assessments, including tactile-visual examination without radiographs were completed by pediatric dentists or pediatric dental program residents, and recorded the surface on a tooth chart. Multiple dental researchers were trained for the data abstraction by an author, MH, and conducted a double data entry to minimize human errors. The two databases were compared to indicate human errors. The errors were corrected in the final database. All data were entered into REDCap Database, an encrypted password protected web based database. The Institutional Review Board of Case Western Reserve University approved this research protocol.

-Measures

Characteristic child information including the child’s age, gender, insurance status (uninsured, private dental insurance, or Medicaid), the total number of primary teeth present, and the number of primary teeth with untreated dental caries (i.e. dt) were abstracted, along with geographic information, the name of city. The number of dentists who were identified by acceptance of Medicaid (i.e. Medicaid dentist) was collected from the website of the Ohio Department of Medicaid (http://www.medicaid.ohio.gov). The population, population density (people per kilometer squares), the number of children (under 18 years old) of each city where the Head Start Program site was located were collected to calculate the density of Medicaid dentists (the number of Medicaid dentists per 10,000 children, i.e. Medicaid dentist density) from the United States Census Bureau (http://www.census.gov/en.html). Additionally, whether the cities provided fluoridated water was collected from their water system websites or by telephone interview with the water departments of each city.

-Statistical Analysis

Bivariate analyses were conducted to describe the relationship between children’s demographic and sociogeographic factors with dt. A negative binomial regression model analysis was conducted to test the association of sociogeographic factors with dt. For the negative binomial regression model analysis, the population was divided 1000 in order to adjust its scale with other variables’ scale. All analyses performed by IBM SPSS statistics 24 (IBM© Corp., Armonk, NY, USA).

## Results

Twelve charts that had a critical lack of information were eliminated from the data analyses, leaving 388 charts of which 200 (51.5%) were boys. Eighty-two Head Start sites and 17 cities were identified. The mean (standard deviation, i.e. SD) of children’s age was 3.51 (1.14) years with a range of 7 months to 5 years. Three (0.8%) children were uninsured, 20 (5.2%) were covered by private dental insurance, and 365 (94.1%) were on Medicaid. The mean (SD) of children’s untreated dental caries in primary teeth was 0.84 (2.16) teeth and the range was 0 to 18 teeth.

Bivariate analyses for dt with children’s demographic factors, child’s age, gender, and type of insurance, were conducted. There were no significant differences in the mean of the dt between boys (mean (SD)=0.73 (2.04)) and girls (mean (SD)=0.96(2.28)), (t=-1.08, df=386, *p*=0.279). There were no significant differences in the mean of the dt among types of insurance, uninsured, private insurance, and Medicaid, 0 (0), 1.3 (3.13), 0.82 (2.11), respectively (F=0.69 (2,385), *p*=0.501). On the other hand, a correlation analysis between dt and child’s age indicated a significant relationship (Pearson correlation=0.343, *p*<0.001).

 Table 1 shows the descriptive information by city where the Head Start sites were located. Two of the 17 cities did not have access to fluoridated water. The range of the number of Medicaid dentists was 0 to 449. The range of Medicaid dentist density was 0 to 624.18. Analysis of variance (ANOVA) indicated a trend of differences (F=1.62(16,371), *p*=0.061) in dt among cities.

**Table 1:**
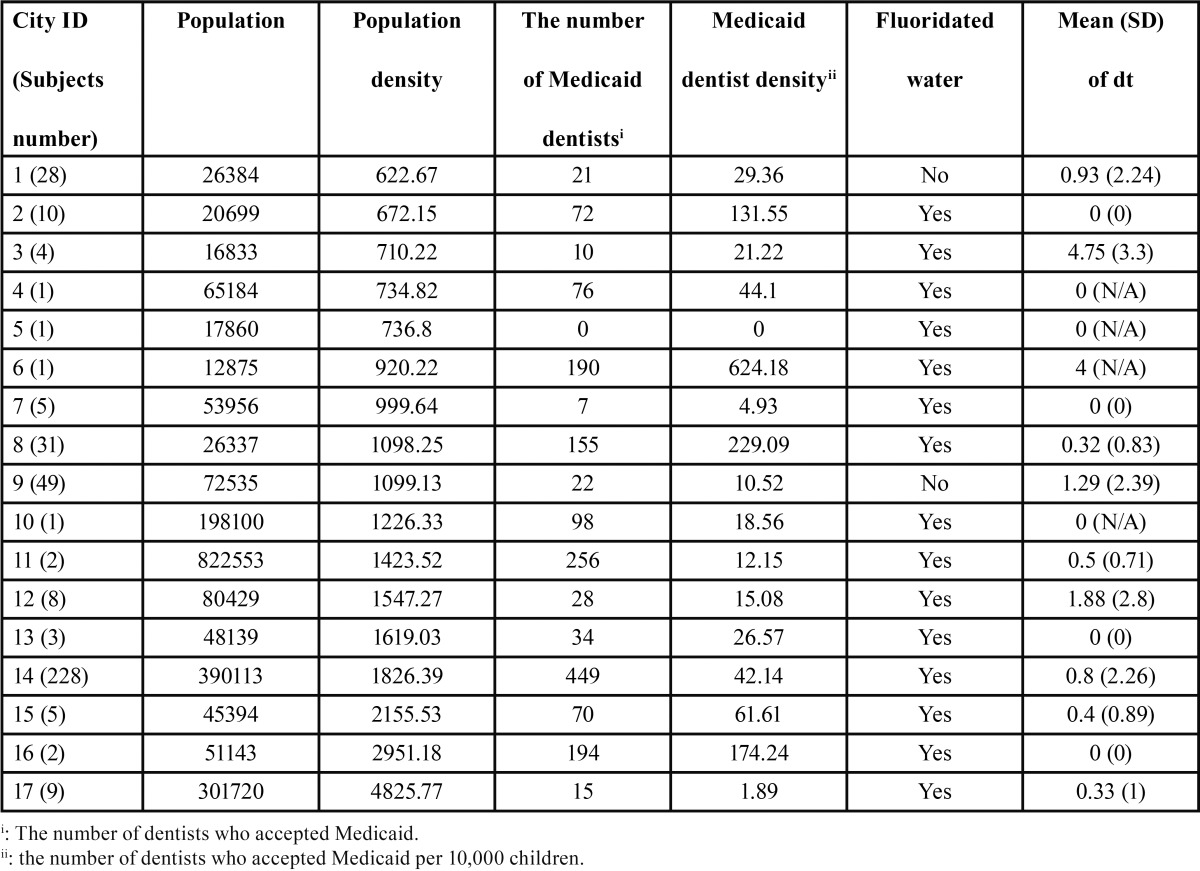
The number of primary teeth with untreated dental caries (dt) and social geographic information by city.

Correlation analyses were conducted among sociogeographic factors, population, population density, total number of Medicaid dentists, and Medicaid dentist density. There were highly significant correlations among the sociogeographic factors (e.g. Peason correlation between population and Medicaid dentists was 0.909, *p*<0.001), except between total number of Medicaid dentists and Medicaid dentist density (Peason correlation=-0.062, *p*=0.226).

Children (n=311) who had available fluoridated water indicated significantly less dt than children (n=77) who did not have it available, 0.76 (2.09) and 2.39 (11.24), respectively (t=2.44, dt=386, *p*=0.015).

The negative binomial regression model was significance (Likelihood Ratio Chi-Square=181.4 df=8, *p*<0.001).  Table 2 shows estimated ß-Coefficients with p-values. After controlling the children’s demographic factors, the density of Medicaid dentists indicated a negative significant association with dt (*p*=0.030). Additionally, being supported by Medicaid also indicated a negative significant association with dt (*p*=0.040).

**Table 2:**
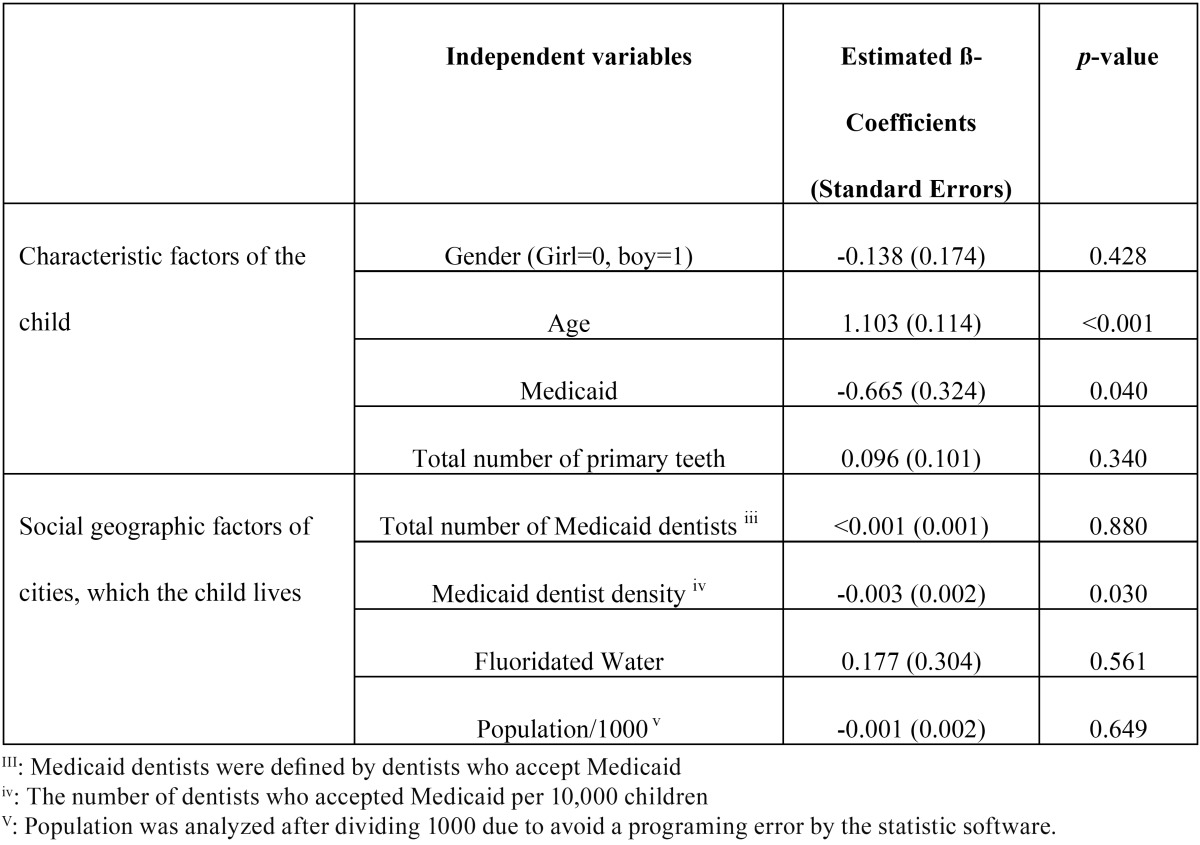
Outcomes of the negative binomial regression model analysis for the number of primary teeth with untreated dental caries.

## Discussion

This retrospective chart review was conducted among children from low-income families to investigate the effect of sociogeographic factors on children’s oral health.

Although the effect of fluoridated water on children’s oral health has been studied (16-18), other sociogeographic factors’ influence on children’s oral health have not been adequately researched yet (12). The effects of sociogeographic factors, such as the density of available dentists, have been tested with children’s dental utilization as an outcome (8,9,11,15). We could not find existing research, which tested the effect of the sociogeographic factors on children’s oral health as a main outcome except the research on fluoridated water. This study helps to understand the association between the social geographic factors and children’s oral health.

Providing fluoridated water is well-known means of preventing children’s dental caries; meta-analysis regarding fluoridated water research indicated a positive benefit for prevention of dental caries in primary teeth. Studies among similar low-income populations (16-18) also indicated that children who had access to fluoridated water showed less dental caries than in children who did not have access to fluoridated water. By bivariate analysis, children who live in cities with fluoridated water indicated significantly less dt compared to children who live in cities without fluoridated water. After controlling other variables by the negative binomial regression model analysis, this significant association has disappeared (Estimated ß-Coefficients (Standard Errors)=0.177 (0.304), *p*=0.561).

The positive effect of available dentist density on children’s dental utilization has been reported (8,9,11,15), along with a positive effect of dental utilization on children’s oral health status (4,5,20). Our study demonstrated a significant association between the density of available dentist who accept Medicaid and children’s oral health status. Children’s dental utilization may play a mediator between the available dentist density and children’s oral health status. To understand more about the relationship among factors, the dentist density, children’s dental utilization, and children’s oral health, a mediation analysis with a bigger sample size will need to be conducted in future research.

To reduce the children’s oral health disparity, the effect of providing fluoridated water is significant for primary prevention. However, there are also disadvantages, such as cost and dental fluorosis (21). In the U.S., the dentists who accept government-assisted insurance is low (14). Although further research is required, increasing the number of dentists who accept government-assisted insurance and connecting them to the children of low-income families is a possible strategy in reducing children’s oral health disparities. As additional information, although not significant, children with private insurance indicated more untreated dental caries than children who have Medicaid in our data, which was a converse outcome from a previous report (22). All cost for dental care for children with Medicaid is paid for by Medicaid, however, children with private insurance have to pay a part of the cost for dental care even though they may benefit from the wider treatment options not covered by Medicaid. Our target population was children from low-income families. Within this population, having private insulance may be a barrier for children’s dental utilization.

There are a few study limitations to this research. Our model did not follow patients’ posible travel into other cities for dental treatment. Also, some dentists who accepted government-supported insurance admitted only adults. We only had the number of untreated teeth and not the number of dental caries experienced teeth, such as filled or extracted teeth due to dental caries. Finally, our findings are generalized to children in the HS programs in the Northeast Ohio area and may not be applied to other populations.

## Conclusions

We observed a negative significant association between the density of available dentists and children’s untreated dental caries among children in Head Start programs in Northeast Ohio. Increasing available dentists may be a strategy to reduce the number of early childhood caries.
